# Elements That Influence the Development of Attention Deficit Hyperactivity Disorder (ADHD) in Children

**DOI:** 10.7759/cureus.27835

**Published:** 2022-08-09

**Authors:** Amina Yusuf Ali, Bithaiah Inyang, Feeba Sam Koshy, Kitty George, Prakar Poudel, Roopa Chalasani, Mastiyage R Goonathilake, Sara Waqar, Sheeba George, Wilford Jean-Baptiste, Lubna Mohammed

**Affiliations:** 1 Pediatrics, California Institute of Behavioral Neurosciences & Psychology, Fairfield, USA; 2 Research, California Institute of Behavioral Neurosciences & Psychology, Fairfield, USA; 3 Internal Medicine, Chitwan Medical College of Medical Science, Chitwan, NPL; 4 Research, California Institute of Behavioral Neurosciences & Psychology, fairfield, USA; 5 Pediatrics/Internal Medicine, California Institute of Behavioral Neurosciences & Psychology, Fairfield, USA; 6 Internal Medicine, California Institute of Behavioral Neurosciences & Psychology, Fairfield, USA

**Keywords:** nutritional deficiency, prenatal risks, hypothyroid, heavy metal toxicity, organic pollution, maternal factors, children, attention deficit hyperactivity disorder (adhd)

## Abstract

Various factors may have a role in the development of attention deficit hyperactivity disorder (ADHD). Although the specific pathophysiology of this disease is still not entirely understood, it is believed to be caused by a mix of genetic, maternal, dietary, and environmental factors. The effect of these factors can determine the severity of ADHD; for example, some of them are dose-dependent, but there is a typical pattern that all are known to be associated with either early childhood exposure or maternal exposure during pregnancy. Some factors share a similar mechanism of affecting pathways and increasing the risk of ADHD.

ADHD is not a disorder that can be detected before symptoms appear in a child, making it more challenging to anticipate even if a child has been exposed to a known trigger. Environmental pollutants were investigated, and it was shown that there was a link between ADHD in childhood and exposure to pollutants throughout childhood or during pregnancy. It is well known that maternal health is a significant factor in the unborn child's development in many health aspects. The central nervous system (CNS) is a primary system that can suffer irreversible damage from health conditions, stress, depression, or specific nutritional deficiency when the pregnant mother is subjected to these conditions. Even though numerous studies have been conducted to investigate the probable causes of ADHD, with some of them having robust findings, no conclusive explanation can be provided to identify a definitive cause or a risk factor.

## Introduction and background

Attention deficit hyperactivity disorder is a neurobiological condition characterized by impaired attention, impulsivity, and hyperactivity that often begins in childhood and frequently persists into adulthood [1]. The idea is well-expressed in this quote by Sarah Young, which says, "living with ADHD is like being locked in a room with 100 televisions and 100 radios all playing. None of them have power buttons so you can turn them off and the door is locked from the outside" [2]. ADHD affects around 5% of children under 18 and 2.5% of adults worldwide [1]. It is also associated with other mental health problems, such as personality disorders and substance abuse. In addition, ADHD can have a negative impact on one's social and personal life, leading to many undesired outcomes, such as educational underperformance, unemployment, and involvement in criminal activities. ADHD is now considered among the most commonly diagnosed chronic psychological disorders in children [1].

Some studies have shown that ADHD is associated with various environmental factors. There are primarily prenatal risk factors such as maternal stress, smoking, drinking alcohol in pregnancy, and perinatal factors such as low birth weight and prematurity, environmental toxins (organophosphates, polychlorinated biphenyls, lead), psychosocial conditions (severe early childhood neglection, aggressive maternal behavior), and nutritional deficiencies. However, it has not been proven that the variables are causal, that they are not distributed randomly among the population, or that the connections detected could also be due to confounding variables or selection bias, as ADHD itself could result in increased exposure to certain environmental factors [3]. Additional environmental factors, such as exposure to audiovisual media and fast-paced television, may contribute to later childhood attention problems [4]. ADHD has been associated with nutrient deficiencies and "unhealthy" eating habits. Not just certain nutrients, but also the complete diet should be considered in ADHD [5]. It was found that maternal deficiencies of iron, zinc, vitamin D, and omega-3 polyunsaturated fatty acids (omega-3) affect the developing brain of the fetus [6-9]. Also, data indicates that all forms of hypertension during pregnancy (HDP), particularly early-onset pre-eclampsia, are related to an increased risk of offspring developing neurodevelopmental disorders. These findings emphasize the critical need for improved maternal HDP care and early detection and screening for children's neurodevelopmental problems [10].

In this traditional review, we will highlight the maternal and environmental factors as well as nutritional deficiencies that could lead to the development of ADHD in children. Identifying these factors will also help focus on prevention and reduce the incidence of ADHD, as it affects many aspects of the lives of children and families and is usually associated with other psychiatric and mental comorbidities.

## Review

Discussion

**Figure 1:**
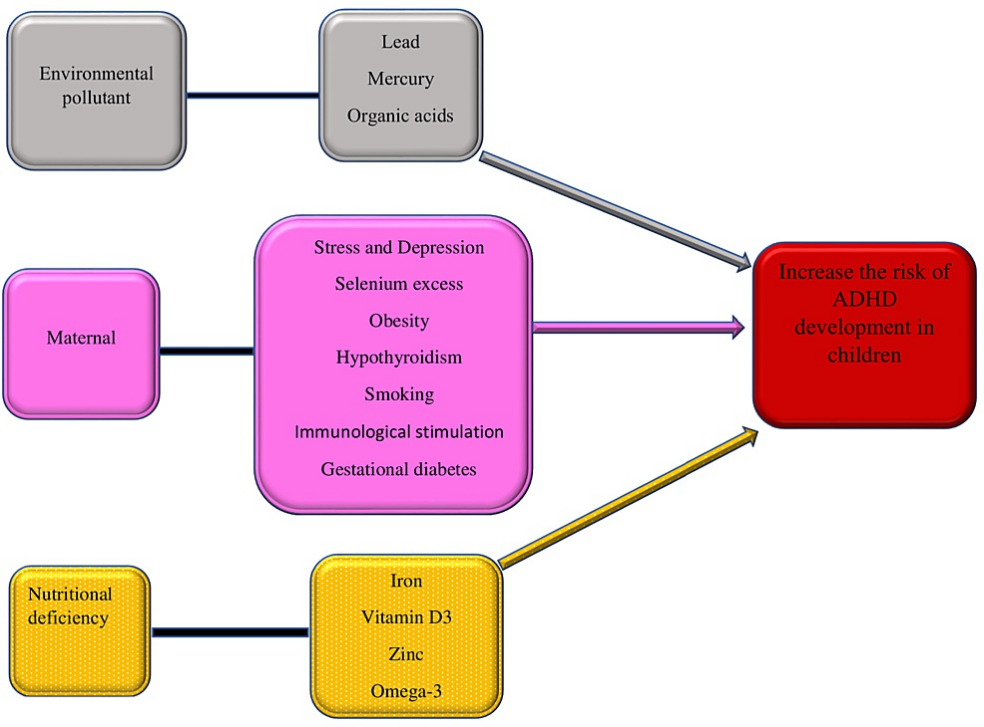
Some of the factors discussed in this review that lead to an increased risk of ADHD development in children. ADHD, attention deficit hyperactivity disorder; omega-3, omega-3 polyunsaturated fatty acids Illustration created by the first author

In this section, we summarized the research and focused on the link between certain factors and the risk of ADHD in children, as shown in Figure 1.

Exposure to Environmental Pollutants

A population-based pregnancy cohort study was conducted by the Norwegian Institute of Public Health. At roughly gestational week 18, blood samples were taken from both parents and the child's umbilical cord at birth to check for concentrations of some organic acids. Perfluorooctanoic acid (PFOA), polyfluoroalkyl substances (PFAS), perfluorodecanoic acid (PFDA), perfluororoundecanoic acid (PFUnDA), and perfluorohexane sulfonate (PFHxS) were measured, and the connection with increased ADHD diagnosis was investigated [11]. These substances are widely used because of their water, oil, and stain-resistant qualities. They are used in frying pan coatings, waterproof clothing, non-stick food packaging polishes, (ski) waxes, paints, cleaning products, and fire-fighting foams. The biggest body burden is from eating contaminated food (mostly fish and processed meats) [12-14]. PFOA exposure during pregnancy was associated with an increased risk of ADHD. The current study's findings indicated an increased risk of ADHD and autism in children exposed to mid-range levels of PFOA during pregnancy and that this connection was non-linear in both case groups [11].

The link between prenatal and postnatal exposure to environmental pollutants such as polychlorinated biphenyl-153 (PCB-153), dichlorodiphenyldichloroethylene (p-p'-DDE), and hexachlorobenzene (HCB), and childhood ADHD was investigated by analyzing PCB-153, p-p-p'-DDE, and HCB concentrations in cord blood, maternal blood, and milk from seven European birth cohort studies. They used the specific pharmacokinetics model to calculate persistent organic pollutant (POP) concentrations at birth, and at 3, 6, 12, and 24 months. That was an extensive study with a sample size of 4,437 children on the link between prenatal and postnatal POP exposure and ADHD development in the general population. It has been found that prenatal and early postnatal exposure to PCB-153, p-p'-DDE, and HCB did not raise the risk of ADHD in a sample of 4,437 children from seven European birth cohort studies. Future research should combine psychosocial, genetic, and epigenetic data with information about environmental pollutants to assess if environmental toxicant exposure contributes to ADHD prevalence [15].

Another study showed that 10 chemical exposures were associated with ADHD. Brominated diphenyl ether (BDE) 47 and 154, PFOA, PFOS, polychlorinated biphenyl-114 (PCB-114), and -HCH were associated with an increased risk, while BDE-153, PCB-153, HCB, and p, p′-DDT were associated with a decreased risk. The results for PFOS, HCB, β-hexachlorocyclohexane (β-HCH), and p, p′-DDT were the most solid. A prospective Norwegian cohort showed that PFOS and β-HCH were related to an increased risk, while p, p′-DDT were discovered to be related to a lower risk, and HCB exhibited a non-linear exposure-response relationship. These pollutants are organic substances, and their body burdens will remain high for the foreseeable future due to their extended half-lives and environmental persistence. In this study population, fish consumption was substantially associated with levels of PFOS, β-HCH, HCB, and p, p′-DDT. This finding corroborates previous findings demonstrating that fish consumption is the primary source of dietary exposure to these four POPs. A surprising and significant association was noted between increasing β-HCH concentrations in breast milk and an increased incidence of ADHD. No conclusive link between PCBs and ADHD diagnosis was found; the bulk of past studies have indicated null or near-null correlations for PCBs. However, a pooled assessment of PCB-153 in Europe discovered no association with the incidence of ADHD. Additionally, there was no solid evidence linking polybrominated diphenyl ethers (PBDEs) to ADHD found. BDE-47 was shown to be connected with an increased risk of ADHD, but BDE-153 was found to be associated with a decreased risk [16].

Current studies suggest that the neurotoxicity due mercury (MeHg) is caused by a number of well-known processes that target epigenetic regulatory systems such as DNA methylation, oxidative stress, toxicity to mitochondria, and disruption of calcium homeostasis. According to research on the relationship between DNA methylation and prenatal mercury exposure, this exposure may be connected to the pathophysiology of ADHD. In addition, MeHg exposure may be related to the epigenetic control of dopamine-metabolizing genes. Dopamine agonists interact with the dopaminergic system during development to modify the pharmacological effects of dopamine agonists. Dopaminergic neurotransmission is involved in cognitive processes such as the reward system, muscular control, and emotion regulation. The effectiveness of dopamine neurotransmission stimulants in treating ADHD shows that the normal dopaminergic neurotransmission in ADHD patients may have been interrupted [17].

According to the research, an unhealthy diet remains a significant factor in the development of ADHD symptoms, and ultra-processed food consumption may be a source of heavy metal exposure, particularly for inorganic mercury and lead (Pb), both elements are neurotoxic. Low-level exposures to lead, in conjunction with other neurotoxic chemicals, such as mercury and arsenic, have detectable effects on the neurodevelopment of children. Moreover, children with ADHD continue to exhibit higher levels of MeHg or Pb in their blood, which can be identified via blood testing. If blood testing reveals increased levels of mercury or lead, the physician may refer the patient for a dietary evaluation and healthy diet education [18].

Maternal Factors

Maternal inflammation during pregnancy, in particular, may instruct fetal inflammatory pathways and epigenetic machinery, potentially increasing the expression of neurodevelopmental problems in childhood. The magnitude, kind, intensity, and timing of the immune response during pregnancy likely affect fetal neurobehavior via their effects on the developmental time courses of various brain cells, regions, and underlying neural processes. In epidemiological research, it has been challenging to establish a link between the timing of exposure to risk variables and childhood neurodevelopmental disorders [19].

Smoking throughout the first trimester appears to enhance the risk of developing ADHD. Prenatal maternal smoking was found to be related to ADHD in offspring. Mothers who smoked heavily (OR: 1.75 [1.51-2.02]) had a significantly elevated risk of ADHD in their offspring compared to mothers who smoked lightly (OR: 1.54). Maternal obesity was related to an increased risk of ADHD (RR: 1.64 [1.57-1.73] versus overweight status (RR: 1.28 [1.17-1.40], implying a dose-dependent increase in risk. Maternal sadness was found to be unrelated to childhood ADHD [19].

Prenatal stress exposure (PNSE) has been extensively linked to preterm delivery and intrauterine growth restriction. PNSE can also be directly assessed using questionnaires, clinical interviews, and biological samples, such as cortisol from maternal saliva, blood, or amniotic fluid, collected prospectively from samples of pregnant women. Prenatal stress alters the development of the amygdala and hippocampus and influences the production and transport of dopamine and the expression of critical receptors. The dopaminergic system serves a variety of activities essential to temperament, including prioritizing stimuli or responses and aiding alternative selection in either a perceptual-cognitive or motor-behavioral setting. The serotonin signaling system also has relevance for temperament, given its association with depression and anxiety associated with negative emotions and has significant consequences for temperament, particularly stress reactivity, fear and anxiety, impulsivity, and attention/regulation [20,21].

ADHD was found to be substantially related to maternal autoimmune disorders and asthma. Children born to mothers who experienced asthma exacerbations had a significantly increased risk of ADHD (HR: 1.25 [1.08-1.44]), implying that a more severe asthma phenotype results in a higher risk for the offspring. Maternal immunological stimulation may serve as a precursor for neurological diseases via activated microglia. Microglia with altered morphology and density and enhanced microglial gene transcription have been identified in brain samples from individuals with various neurodevelopmental problems. Microglia are macrophages in the CNS that play a vital role in neurogenesis, myelination, synaptic pruning, and brain homeostasis. Microglia that have been activated develop a pro-inflammatory phenotype, typically in reaction to infection and in response to cell injury and death in their environment. Microglia are remarkable in that they begin in the yolk sac and rarely replace peripheral cells during normal development. Thus, early-life microglial perturbations have the potential to program microglia into an activated state, thereby increasing their vulnerability to immunological stimuli later in life. As a result, preconditioning microglia by maternal immune activation may increase susceptibility to neurobehavioral disorders in childhood [19].

The relationship between gestational diabetes (GDM) and ADHD was examined. Hyperglycemia may affect neurodevelopment via oxidative stress, which is linked to poor neurobehavioral outcomes such as motor deficits. It may also affect epigenetic changes in kids, such as the decreased DNA methylation observed in neurodevelopmental disorders. Additionally, hyperglycemia can result in systemic inflammation, as pro-inflammatory cytokines can penetrate the placenta and fetal blood-brain barrier, impairing neurodevelopment. However, women with GDM are more likely to experience many unfavorable obstetric outcomes, including pre-eclampsia, fetal macrosomia, perinatal mortality, cesarean section, instrumental delivery, premature delivery, and neonatal asphyxia, all of which may raise the risk of neurodevelopmental problems [22].

The effect of exposure to neonatal asphyxia results in brain damage. Prior research has demonstrated that hypoxia can create long-term abnormalities of the dopaminergic system and even reduce the expression of the dopamine D2 receptor, hence increasing the risk of ADHD. In addition, current research indicates that hypoxia can change catechol-O-methyltransferase (COMT) gene expression; the COMT gene has been linked to ADHD or ADHD symptoms [23].

The effect of maternal thyroid hormone deficiency during early gestation on the development of ADHD was examined. Children exposed to maternal hypothyroxinemia had higher ADHD scores than non-exposed children. There was no correlation between maternal subclinical hypothyroidism and ADHD scores in children. The fetal brain is dependent primarily on circulating T4 levels for intracellular triiodothyronine supply. Hypothyroxinemia during pregnancy results in irreversible cytoarchitecture defects in the sensorimotor cortex and hippocampus [24].

ADHD symptoms are considered to be caused by dysfunctions in the frontal and prefrontal lobes, which regulate impulsivity and motor activity, as well as in the cortical and subcortical striatal areas, which regulate irrelevant responses and executive function. Also, research demonstrated that children with ADHD had abnormalities in the microstructure of the corpus callosum frontal regions and the white matter connections underlying the primary and somatosensory motor cortices. Additionally, thyroid function is required for the correct development of the monoaminergic and cholinergic neurotransmitter systems, and dysfunctions in these transmitter systems have been associated with attention deficiencies and hyperactivity [24]. Both neurons and glial cells have high levels of thyroid hormone receptors. According to research, T3 binds to a thyroid- hormone receptor, initiating gene transcription; this promotes the expression of certain gene patterns implicated in axonal and dendritic expansion, synapse formation, myelination, cell migration, and proliferation of specific cell types. For correct neuronal organization, interaction with glial cells is necessary. Consequently, it is evident that any condition that impedes the passage of maternal thyroid hormone to the fetus would impair neuronal migration. Consequently, neurons will not reach their destination in the top layers, and their aberrant location will affect the laminar architecture of the cerebral cortex [25].

A mother's depressive symptoms during pregnancy are linked to ADHD symptoms in children. At the age of three to six years, children of mothers who experienced persistently high depression symptoms throughout pregnancy demonstrated increased levels of ADHD symptoms. The study discovered that increased maternal depressive symptoms following pregnancy were connected to increased child ADHD symptoms. Existing research indicates that increased maternal depressive symptoms and salivary cortisol levels during pregnancy are associated with changes in the offspring's brain structure, functional connections, and right hemisphere cortical thinning. Symptoms of depression during pregnancy have also been associated with increased mRNA levels of placental glucocorticoid and mineralocorticoid receptor genes, implying an increased glucocorticoid sensitivity in the placenta. Emerging evidence suggests that inflammatory markers may be involved, as maternal depressive symptoms and pro- and anti-inflammatory cytokines during pregnancy have been linked. Early pregnancy screening and preventive interventions targeting maternal depressive symptoms may benefit maternal and offspring well-being [26].

According to available literature, both selenium (Se) deficiency and excess may be harmful to health. Currently, the function of maternal Se status in the long-term neurodevelopment of children is largely unknown. The study examined the temporal and dose-response relationships between maternal Se status and the risk of children's neurodevelopmental disorders such as ADHD. Se levels in maternal RBCs were determined in samples taken within 72 hours of delivery. Analyses of the data revealed a positive correlation between maternal Se and ADHD (OR: 1.29; 95% CI: 1.04-1.56, per IQR increase in Se). Even after correcting for relevant covariables, these relationships remained robust, and there was no significant interaction between Se and these covariables. Our data indicate that prenatal exposure to elevated maternal serum Se levels may have a detrimental effect on child neurodevelopment [27].

The Effect of Some Nutritional Deficiencies

A study was performed measuring maternal 25-hydroxycholecalciferol (25(OH)D). The mean age at ADHD diagnosis was 7.3 years. The median maternal 25(OH)D level was 29.2 nmol/L. The mean gestational age when samples were taken was 10.7 weeks. This was the first study to link low maternal 25(OH)D levels in early to mid-pregnancy to ADHD in children. Two studies relate low maternal 25(OH)D levels to early pregnancy, supporting this result. Inadequate vitamin D in pregnancy may disrupt fetal programming and expose the child to a suboptimal environment, perhaps contributing to ADHD. Vitamin D affects brain function through controlling calcium signaling, neurotrophic and neuroprotective effects, neuronal differentiation, maturation, and growth. Aberrant attention processing causes alterations in dopamine system ontogeny, novelty-induced hyperlocomotion, and latent inhibition. The risk of ADHD was considerably increased in toddlers with maternal and neonatal vitamin D deficiency [6,28].

A zinc deficiency effect on the development of ADHD was examined; more than 200 zinc metalloenzymes are needed for neuronal proliferation. Changes in dopamine and serotonin neurotransmitters may explain the link between zinc and ADHD. Zinc is needed for the creation and control of melatonin, which regulates dopamine function, and for converting dietary pyridoxine to pyridoxal phosphate, which converts tryptophan to serotonin. ADHD affects the dopamine and serotonin systems. Zinc may counteract melatonin and serotonin declines and relieve ADHD symptoms. In several countries, blood zinc levels were lower in children with ADHD than in controls, suggesting a link to symptom severity. Zinc tissue concentrations (e.g., serum, red blood cells, hair, urine, and nails) are lower in children with ADHD than in normal control subjects and compared to population norms [7].

Children born to mothers with iron deficiency anemia diagnosed at 30 weeks or less were more likely to be born preterm (OR: 7.10; 95% CI: 6.28-8.03) or small for gestational age (OR: 2.81; 95% CI: 2.26-3.50), whereas children whose mothers were diagnosed with anemia at greater than 30 weeks gestation were more likely to be born post-term and large for gestational age. Anemia identified in 30 weeks or less of pregnancy was linked to a greater incidence of ADHD in offspring. These data show the importance of early iron testing and dietary counseling during prenatal care [8].

A meta-analysis study shows that teens with ADHD had low docosahexaenoic acid (DHA). Lower maternal intake of omega-3 polyunsaturated fatty acids (omega-3 or n-3 PUFAs) during pregnancy is connected to poorer developmental outcomes in the offspring, such as lower scores on fine motor, communication, and social development assessments. Developmentally disabled children have low DHA. Although omega-3 insufficiency may be a cause of ADHD, approximately 20-40% of ADHD patients do not benefit from pharmacotherapies such as methylphenidate and atomoxetine [26]. Children of mothers with a poor seafood diet during pregnancy are at risk of prosocial behaviors, fine motor coordination, linguistic communication, and social development. Children with ADHD had low plasma DHA levels. In clinical trials, n-3 PUFAs improved ADHD symptoms and cognitive function in clinical trials. Some children see no benefit at all [9]. Seafood consumption during pregnancy should be carefully monitored to avoid the risk of mercury toxicity and its effect on the fetus. One study showed that omega-3 fatty acids (fish oil) supplementation during early pregnancy raised the incidence of schizophrenia and ADHD symptoms [29].

## Conclusions

ADHD and other neurodevelopmental problems have a wide range of consequences for children and their families, yet relatively few studies on the subject are conclusive. Knowing the exact variables and ways of exposure that create ADHD would be excellent for preventing ADHD. There are no definitive therapies available yet, only symptomatic medicines that do not manage symptoms entirely in many cases; therefore, understanding the mechanism of development and risk factors contributing to the development of the condition is essential.

More concentrated retrospective research including bigger groups of individuals is required to gain a good understanding of the causative elements rather than just the risk factors that are involved. It is still not fully understood why some children develop ADHD and some do not; even if they were exposed to the same factors, there might be protective variables that need to be researched. In addition, we had difficulties locating much research conducted in the past that investigated the risk factors, and the studies we found had a small number of subjects.
